# DREAM Mediated Regulation of GCM1 in the Human Placental Trophoblast

**DOI:** 10.1371/journal.pone.0051837

**Published:** 2013-01-03

**Authors:** Dora Baczyk, Mark Kibschull, Britt Mellstrom, Khrystyna Levytska, Marcos Rivas, Sascha Drewlo, Stephen J. Lye, Jose R. Naranjo, John C. P. Kingdom

**Affiliations:** 1 Research Centre for Women's and Infants' Health at the Samuel Lunenfeld Research Institute, Mount Sinai Hospital, University of Toronto, Toronto, Ontario, Canada; 2 Department of Molecular and Cellular Biology, National Centre for Biotechnology, C.S.I.C., Madrid, Spain; 3 Department of Obstetrics and Gynecology, Mount Sinai Hospital, University of Toronto, Toronto, Ontario, Canada; Brigham and Women's Hospital, United States of America

## Abstract

The trophoblast transcription factor glial cell missing-1 (GCM1) regulates differentiation of placental cytotrophoblasts into the syncytiotrophoblast layer in contact with maternal blood. Reduced placental expression of GCM1 and abnormal syncytiotrophoblast structure are features of hypertensive disorder of pregnancy – preeclampsia. *In-silico* techniques identified the calcium-regulated transcriptional repressor – DREAM (Downstream Regulatory Element Antagonist Modulator) - as a candidate for GCM1 gene expression. Our objective was to determine if DREAM represses GCM1 regulated syncytiotrophoblast formation. EMSA and ChIP assays revealed a direct interaction between DREAM and the GCM1 promoter. siRNA-mediated DREAM silencing in cell culture and placental explant models significantly up-regulated GCM1 expression and reduced cytotrophoblast proliferation. DREAM calcium dependency was verified using ionomycin. Furthermore, the increased DREAM protein expression in preeclamptic placental villi was predominantly nuclear, coinciding with an overall increase in sumolylated DREAM and correlating inversely with GCM1 levels. In conclusion, our data reveal a calcium-regulated pathway whereby GCM1-directed villous trophoblast differentiation is repressed by DREAM. This pathway may be relevant to disease prevention via calcium-supplementation.

## Introduction

Successful placentation in humans is dependent upon the promotion of maternal blood flow to the implantation site and the elaboration of chorionic villi that mediate maternal-fetal exchange [1]. Failure of either or both processes during the early stages of placental development can have negative consequences for both the fetus (intrauterine growth restriction [IUGR]) and the mother, severe early-onset preeclampsia (sPE), causing death or severe long-term morbidity [2].

Human placental development is directed by two distinct lineages of trophoblast cells. Invasive extravillous cytotrophoblast transform the uteroplacental vasculature [3] while villous cytotrophoblast (VCT) give rise to the outer syncytiotrophoblast (SYN) layer that covers the placental villi [4]. The SYN layer is in direct contact with maternal blood and is responsible for maternal-fetal gas, nutrient and waste exchange. Progressive growth of the placental villi during gestation, to accommodate exponential growth of the fetus, requires an accumulation of SYN [5]. This is achieved by continuous proliferation of a subset of VCT [6] that generate post-mitotic cells capable of syncytial fusion [7]. Conserving a population of proliferative VCT throughout gestation is a key feature of human placentation [6] implying that a population of lineage-restricted progenitor cells remains capable of generating a continuous stream of daughter cells to continuously fuse into the overlying SYN.

Development of the mouse labyrinthine trophoblast, analogous to VCT and SYN in the human placenta, is characterized by cell cycle arrest and cell-cell fusion promoted by the transcription factor Glial cell missing-1 (Gcm1) [8]. Mouse embryos lacking Gcm1 die in mid-gestation due to a failure of SYN differentiation [8], [9]. In humans, the homolog GCM1 has an expression pattern similar to mice [10] and plays a central role in mediating differentiation of trophoblast cells along both villous and extra-villous pathways [11]. GCM1 promotes the expression of the fusogenic protein syncytin [12], [13] and placenta growth factor (PlGF) [14].

The trophoblast layer of sPE placentas delivered prematurely shows severe depletion of proliferating VCT, accompanied by patchy necrosis or accelerated apoptosis in the outer SYN layer and SYN knot formation [15]. In parallel, molecular pathology studies have demonstrated reduced expression of GCM1 [16] and its downstream fusogenic partner syncytin [17] in sPE placentas while low maternal circulating PlGF levels are predictive of the severity of this disease [18]. Inhibition of GCM1 expression in the first trimester floating explant model *in-vitro* recapitulates these SYN abnormalities observed in sPE [11].

GCM1 is therefore a key regulator of human placental development, though the mechanisms governing its regulation are poorly understood. To explore potential upstream molecular mechanisms of GCM1 transcriptional regulation we conducted an *in silico* analysis of the GCM1 promoter region that revealed the existence of DREAM binding sites.

DREAM (also known as KChIP-3 and calsenilin) belongs to a family of small calcium sensors that are calcium-dependent transcriptional repressors [19], [20]. DREAM contains 256 amino acids and 9 exons (molecular weight of 29 kDa); the human placenta only expresses a specific splice variant containing 230 amino acids and 8 exons [21]. Both isoforms encode a protein containing four calcium-binding domains [19]. DREAM is expressed in different cellular compartments (nucleus, cytoplasm and cell membrane) and thus is involved in various distinct cell functions. Nuclear accumulation and thus repressor function of DREAM has been related to sumoylation of the protein [22]. DREAM is a multifunctional protein critical for brain function since i) regulates calcium homeostasis and neuronal viability [23], ii) interacts with presenilins, Alzheimer's disease- associated proteins, to regulate APP processing [24], [25] and iii) regulates neuronal excitability by modulating potassium and calcium channels [26], [27] as well as NMDA receptors [28] [29]. We hypothesized that in the human placenta, DREAM mediates the expression of GCM1 via a direct interaction with its promoter. Here we demonstrate that DREAM negatively regulates GCM1 gene expression during human placental development in a calcium-regulated manner and we show that sumoylated DREAM is increased in sPE placentas. These findings may be of relevance to clinical trial observations that show calcium supplementation reduces the risk of this disease in women with low calcium diets [30].

## Materials and Methods

### BeWo cells

The human choriocarcinoma cell line BeWo was purchased from ATCC, (Manassas, VA). Passages 10–20 were used for the experiments. The BeWo cell line was maintained in F12K medium (ATCC) supplemented with 10% FBS, 100 units/ml penicillin, 100 units/ml streptomycin, and 2.5 µg/ml fungizone (Invitrogen, Burlington, ON), in atmospheric O_2_ and 5% CO_2_ at 37°C.

For DREAM siRNA treatment and DREAM over-expression assays, 100,000 BeWo cells per well in 1 ml of regular media were plated into 12 well plates (Sarstedt, Montreal, QU) 24 hours prior to transfection. Transfections were performed in serum and antibiotic free OPTI MEM medium (Invitrogen). 50 nM of DREAM siRNA, 50 nM non-silencing control (Cat # sc-42398, sc-37007; Santa Cruz Biotechnology, Santa Cruz, CA) or 1 µg of DREAM (short isoform, NM_001034914.1) over-expression plasmid (pcDNA3.1 V5-His-TOPO vector; kind gift from Dr. Timmusk, Tallinn, Estonia) [21] was transfected into the cells with 2 µl of Lipofectamine transfection reagent (Invitrogen) in a total volume of 260 µl for 5 hrs after which time 500 µl of F12K medium with 20% FBS were added. Twenty four hours post transfection, the medium was replaced with 1 ml of regular media. At the conclusion of the experiments, RNA was extracted from the cells using RNeasy Mini kit (Qiagen, Mississagua, ON) according to the manufacturer's recommendations.

### Tissue collection

Placental villous samples from first and second trimester were obtained from Morgentaler Clinic, Toronto, Canada, following a voluntary legal termination of pregnancy. Mount Sinai Hospital (MSH) Research Ethics Board approval (MSH REB #04-0018-U) was obtained for this study and all patients gave written informed consent. Gestational age and viability were established pre-operatively by ultrasound. All experiments were established within 4–6 hrs from the time of sample collection.

Individual clusters of 8–12 week gestation villi (20–30 mg wet wt) were dissected in sterile cold PBS containing calcium and magnesium, under a microscope. The proximal stem villi were inserted into the underside of sterile polystyrene cubes so as to float the villous trees in 750 µl of serum-free media (DMEM/F12) with 1% liquid media supplement ITS+1 (Sigma, St Louis, MO, USA), 100 units/ml penicillin, 100 units/ml streptomycin, 2 mM L-glutamine, 100 µg/ml gentamycin and 2.5 µg/ml fungizone. These explants were maintained in physiologic 8% ambient oxygen (40 mmHg)/5% CO_2_ at 37°C [11], [31].

For DREAM siRNA treatment, floating villous explants were incubated in the presence of 100 nM siRNA or 100 nM non-silencing control (Santa Cruz Biotechnology) for up to 2 days.

First and second trimester placental samples were also collected into 2 ml of RNA Later buffer (Ambion, Streetsville, ON) and stored at −20°C. Third trimester placental samples, from healthy and severely preeclamptic patients delivering at MSH, Toronto, Canada were collected into RNA later buffer or snap frozen and stored −70°C for future RNA isolation using Qiagen kit. Patient information is summarized in Table 1. Severe pre-eclampsia was defined according to accepted criteria developed by the American College of Obstetricians and Gynecologists (PMID: 16175681).

**Table 1:** Patient Characteristics.

	Gravidity	Parity	Maternal age (yrs)	Race			Delivery mode			Protein-urea	Blood pressure (mmHg)		GA (weeks)	Birth weight (g)	Placental weight (g)
				Black	White	Other	Vaginal	CS with labor	CS no labor		Systolic	Diastolic			
PTL (n = 6)	2.00±0.45	0.50±0.34	30.7±2.7	0	5	1	2	3	1	+0.3±0.2†	120±4	71±2	31.74±0.96	1821.5±133.8	271.2±20.2
PE (n = 8)	1.38±0.18	0	28.4±3.2	2	4	2	0	4	4	+3.8±0.3‡	176±4	111±4	29.23±0.58	1046.4±84.3	185.7±27.1

Values represented as mean+\−SEM. Proteinurea was measured by the dipstick method, with “+4” being the highest value and “0” considered as an absence or undetectable levels of protein.

† All pre-term controls had protein levels of “+1” or lower.

‡ All preeclamptic patients had readings of “+2” or higher, with 7 out of 8 having protein levels recorded as “+4.” GA, gestational age; CS, caesarean section; PE, preeclampsia; PTL, pre-term labor.

### Cytotoxicity assays

Cellular toxicity in treated explants and BeWo cells was assessed with CytoTox96 assay (Promega, Nepean, ON) measuring LDH release into the media. No significant differences were found between and treated and control groups (not presented).

### Bio-Bank Tissue Microarray

Human specimens were obtained by the Research Centre for Women's and Infants' Health (RCWIH) BioBank program of MSH using procedures approved by the MSH Research Ethics Board (MSH REB #10-0128-E). Immediately after delivery, four 2–3 cm^2^ tissue cores through the full thickness of the placenta were obtained from a site within each quadrant, avoiding areas with obvious evidence of thrombosis or other abnormalities when possible. Chorionic plate tissue was also excluded. Cores were rinsed briefly in chilled PBS to remove residual blood and further dissected to generate 0.5–1 cm^3^ pieces. Samples were immersion fixed (4% paraformaldehyde in PBS, 24 h at RT), and paraffin-embedded. The RCWIH BioBank TMA #1 array (http://biobank.lunenfeld.ca/?page=Tissue%20arrays) contained 8 subjects with sPE and 6 age-matched preterm controls. The array was constructed by the MSH Pathology & Lab Medicine Department using a Beecher Instruments Manual Tissue Arrayer.

### Immunohistochemistry

Para-formaldehyde-fixed (4%) placental tissue was wax embedded. Immunohistochemistry was performed on rehydrated sections with the streptavidin-biotin staining procedure, using Peroxidase Dako LSAB kit (Dako Canada Inc. Mississauga, ON, Canada). Primary antibodies to DREAM (clone FL-214, Santa Cruz) and Ki67 (clone SP6, Neo Markers, Fremont, CA, USA) were applied to sections overnight at 4°C. Specific biotinylated secondary antibodies or anti-rabbit IgG2b-AlexaFlur 488 were applied to sections for 1 hr at RT. For immunofluorescence, the slides were treated with 0.4% DAPI for nuclear detection. Fluorescence images were collected using a DeltaVision Deconvolution microscope (Applied Precision, LLC, Issaquah, WA, USA).

Immunohistochemistry for BrdU (10 µM) incorporation on floating villous placental explants was performed according to the manufacturer's instruction by Pathology and Laboratory Medicine at MSH, Toronto, Canada using rat-BrdU antibody (Abcam) and donkey anti-rat IgG F(ab) secondary antibody (Research Diagnostics). A proliferation index was calculated by dividing the number of BrdU-positive villous trophoblast nuclei by the total number of villous trophoblast nuclei counted in five adjacent fields of a 1 cm graticule.

Negative controls included omission of the primary antibody and use of isotype matched Ig. Slides were counterstained with hematoxylin (Sigma), visualized using a Nikon DMRX light microscope and photographed using a Sony PowerHAD 3CCD color video camera DXC-970ND (Sony, Toronto, ON, Canada).

For semi-thin histology, explants were collected into 2% glutaraldehyde and processed by Pathology and Laboratory Medicine at MSH, Toronto, Canada, where they were embedded, sectioned (1 µm) and stained with toluidine blue.

TUNEL labeling was performed in the Pathology Unit at the Centre for Modeling Human Diseases, Toronto, ON, Canada utilizing 10× One-Phor-All buffer and TdT enzyme, FPLCpure ™ (Amersham) according to manufacturer's instructions.

### Reverse transcription and qRT-PCR

Total RNA was extracted from BeWo cells or placental explants and reverse transcribed as described elsewhere [11]. Real time PCR was performed on an Eppendorf epgradientS Master Cycler, in triplicates in 15 µl volumes containing 10 ng of template cDNA, 7.5 µl of 2× SYBR Green PCR Master Mix and 50 nM of primers. The PCR program was initiated at 95°C for 2 min, followed by 40 thermal cycles of 15 seconds at 95°C, 60 seconds at 60°C. A melting curve for primer validation and a template standard curve were performed to show template independent amplification results. To visualize amplification products PCR reaction cocktails were electrophoresed on 1.5% agarose gel in Tris-acetate/EDTA buffer. As a negative control, cDNA template was omitted from the PCR reaction. Comparative C_T_ Method (ABI technical manual) was used to analyze the real time PCR. The expression of DREAM and GCM1 genes was normalized to the geometric mean of SDHA and TBP genes [32] and expressed as fold change relative to non-silenced (NS) control or pre-term placenta.

Intron-spanning primers were designed specifically for human GCM1 and DREAM using Primer Express 2.0 software. TBP and SDHA sequences were obtained from Bieche et al. [32], Hrk primers were a kind gift from Dr. A. Jurisicova (Toronto); all sequences used are shown in Table 2.

**Table 2:** Quantitative real time-PCR primer sequences.

Gene Name		Primer Sequences
GCM1	Forward	5′-GGCACGACGGACGCTTTAT-3
	Reverse	5′-GCTCTTCTTGCCTCAGCTTCTAA-3′
DREAM	Forward	5′-TGGAAGATAGCATCGACATTG-3′
	Reverse	5′-AAGCCCCTGTAGAGAGACTGC-3′
hrk	Forward	5′-CAGGCGGAACTTGTAGGAAC-3′
	Reverse	5′-TCTCCAAGGACACAGGGTTT-3′
SDHA	Forward	5′-TGGGAACAAGAGGGCATCTG-3′
	Reverse	5′-CCACCACTGCATCAAATTCATG-3′
TBP		Sequences are described in [32]

### DNA/protein interactions

The 5000 bp region encompassing the human GCM1 promoter was subjected to analysis by Gene2Promoter Software (Version 6.3, Genomix, Germany) to identify potential transcriptional factor binding sites. Detailed analysis of the promoter revealed numerous DRE core sequences – GTCA.

### Chromatin Immunoprecipitation (ChIP)

DREAM protein and GCM1 promoter interactions were investigated with the aid of a commercial ChIP assay kit (Upstate, CA, USA; catalog # 17-371). The assay was performed according to the manufacturer's instructions with some modifications as described below. DREAM over-expressing BeWo cells and DREAM siRNA treated cells were grown to confluency. Cells were then treated with 1% freshly prepared formaldehyde (Fisher) for 10 min at RT to crosslink the proteins to DNA. Following crosslinking, PBS-washed cells were lysed in SDS lysing buffer and sonicated on wet ice (10 sets of 20-second pulses using High Intensity Ultrasonic Sonicator, 50-watt model, set to 25% of maximum power), resulting in chromatin fragments with a maximum size of 700 bp. One percent of the sonicated DNA/protein fraction was saved as an Input control for further analysis, while the remainder was subjected to immunoprecipitation with: 1) His-tag antibody (Santa Cruz), 2) DREAM isoform 2 specific antibody (Millipore, MS), as well as 3) RNA polymerase antibody (positive control) and 4) rabbit IgG (negative control). Following a pull-down with Protein G Agarose beads, DNA/protein complexes were eluted from the beads and reversed crosslinked under high salt and high temperature conditions (65°C for 5 hrs). Proteins were degraded with Proteinase K treatment for 1.5 hrs and finally DNA was purified using the provided spin columns. The purified DNA was used as a template for PCR reactions. Anti-RNA polymerase fraction was amplified with supplied primers (positive control) and Input DNA and IgG immunoprecipitated fractions were amplified with experimental primers (Table 3).

**Table 3:** PCR primer sequences used in ChIP analysis.

Amplicon Number		Primer Sequences	Amplicon Size (bp)
1	Forward	5′- GATCACCTGAGGTCAGGAGT-3′	527
	Reverse	5′-CAGACTGTCCCATGCACAGAA-3′	
2	Forward	5′-GATAGGGATGCTGTATGATCA-3′	532
	Reverse	5′-GTGTGACATGATGTCCAGACTAG-3′	
3	Forward	5′-GACATGCCTGACATGTTAACAGT-3′	634
	Reverse	5′-TCGACAGGCAATAAGTCAATCAC-3′	
4	Forward	5′-AGCTACTCAGGAGGCTGAGGCA-3′	343
	Reverse	5′-CAATATCCTGGACAACATGACG-3′	
5	Forward	5′-GCAATCTCAGCTCACGCAACCT-3′	781
	Reverse	5′-GAGCAGACGCTGTTCCCTATTC-3′	

One microliter each of Input fraction, RNA polymerase immunoprecipitated fraction, DREAM and IgG immunoprecipitated fractions was subjected to PCR using either provided (positive control) or designed primers. PCR reactions were set up in total 20 µl volume with 2× complete PCR cocktail mix (Fermentas, Burlington, ON) and 0.5 µM of appropriate primers. The PCR program was initiated at 95°C for 3 min followed by 30 cycles of 95°C for 20 sec, 59°C for 30 sec and 72°C for 30 sec and final extension of 2 min at 72°C.

### Electrophoretic Shift Assay (EMSA)

**Figure 1:**
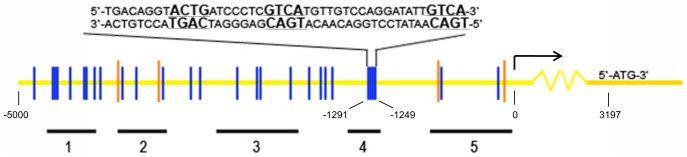
Diagrammatic representation of a 5000 bp fragment encompassing the human GCM1 promoter as well as the transcription and translation initiation sites. Orange bars indicate Gene2Promoter software identified DREAM binding sites (Pu/CNGTCAPuPuPu/C). Blue bars show all putative core DRE sites (GTCA). The transcription initiation site is denoted with an arrow. The translation initiation site is represented by ATG (3197 bp from the transcription initiation site). The enlarged 43 bp sequence, located about 1250 bp upstream from the transcription initiation site is responsible for DREAM binding to the GCM1 promoter. The promoter region was amplified with 5 sets of primers (1–5) during ChIP analysis (See Figure 3). The locations of the amplicons are depicted with the vertical black lines.

Five PCR amplicons from ChIP analysis (Table 3, Figure 1) were 3′ end biotin labeled using a kit (Pierce, Rockford, IL) according to the manufacturer's instructions. Briefly 100 nM of DNA was biotin labeled with enzyme terminal deoxynucleotidyl transferase at 37°C for 30 min. Shorter, 43 bp, 3′ end biotin labeled probe and 5 probes containing mutations in the DRE core sequences were purchased (IDT, Skokie IL) (Table 4).

**Table 4:** 3′ end biotin-labeled probes used in EMSA.

Probe name	Probe sequence (5′ – 3′)
Control	TGACAGGT**ACTG**ATCCCTC**GTCA**TGTTGTCCAGGATATT**GTCA**ACTGTCCA**TGAC**TAGGGAG**CAGT**ACAACAGGTCCTATAA**CAGT**
Mutation 1	TGACAGGT**ACTG**ATCCCTC**GTCG**TGTTGTCCAGGATATT**GTCA**ACTGTCCA**TGAC**TAGGGAG**CAGC**ACAACAGGTCCTATAA**CAGT**
Mutation 2	TGACAGGT**ACTG**ATCCCTC**GTCG**TGTTGTCCAGGATATT**GTCG**ACTGTCCA**TGAC**TAGGGAG**CAGC**ACAACAGGTCCTATAA**CAGC**
Mutation 3	TGACAGGT**GCTG**ATCCCTC**GTCA**TGTTGTCCAGGATATT**GTCA**ACTGTCCA**CGAC**TAGGGAG**CAGT**ACAACAGGTCCTATAA**CAGT**
Mutation 4	CGACAGGT**GCTG**ATCCCTC**GTCG**TGTTGTCCAGGATATT**GTCG**GCTGTCCA**CGAC**TAGGGAG**CAGC**ACAACAGGTCCTATAA**CAGC**
Mutation 5	CGATAGGT**GCTT**ATCCCTCT**TCGT**GTTGTCCAGGATATT**TTCG**GCTATCCA**AGAA**TAGGGAG**AAGC**ACAACAGGTCCTATAA**AAGC**

Core DRE sequences are shown in bold; introduced mutations, in red.

Nuclear proteins were extracted from BeWo cells either treated with DREAM siRNA or over-expressing DREAM his-tagged plasmid. Cells were collected into a buffer containing 25 mM HEPES, 5 mM KCL and 0.5 mM MgCl_2_ solution and then lysed while rotating for 15 min in a buffer containing 25 mM HEPES, 5 mM KCL and 0.5 mM MgCl_2_ and 1% NP-40 resulting in a cytoplasmic protein preparation. To obtain the nuclear protein fraction, the pellet was further lysed for 1 hr at 4°C in a buffer containing 25 mM HEPES, 10% (w/v) sucrose, 350 mM NaCl and 0.01% NP-40. Protein amount was quantified using the Bradford method.

BeWo cell nuclear fraction (5 µg) was incubated with biotin labeled human GCM1 promoter probes (50 ng) for 30 min at RT in a 10 µL volume reaction containing 0.1 µg of Poly d(I-C) and binding buffer (50 mM Tris-HCl (pH 8.0), 750 mM KCL, 2.5 mM EDTA, 0.5 Trition-X 100, 62.5% glycerol and 1 mM DTT). For competition, unlabeled probes were added to the reaction. For supershift with anti- DREAM and anti-HIS tag antibodies, 0.5 µg of the antibody were added to the reaction before adding labeled probe and incubated for 30 min at RT. Five percent non-denaturing TE-polyacrylamide gel and 0.5× TE buffer were cooled at 4°C and the gel was run at 120 V for 30 min prior to loading the gel for equilibration. Protein/probe mix was run at 100 V in the cold room at 4°C for 2 hrs.

Gel components were transferred to a nylon membrane (NEN Life Science Products, Boston, MA) using a semi-dry transfer apparatus (OWL HEP-1, Thermo Scientific) at 200 mA for 1 hr. Following the transfer, the bound probes were immobilized by exposing the membrane to UV crosslinking. Detection of the biotin probes was accomplished with Chemiluminescent Nucleic Acid Detection Module according to the manufacturer's instructions (Pierce) and subsequently exposed to X-ray film.

### Transgenic Mice

DREAM-deficient mice [33] as well as DREAM-over-expressing mice [34] were maintained on a C57BL/6 genetic background under standard housing conditions with a fixed 12-h/12-h light/dark cycle and food *ad libitum* at the Spanish National Center of Biotechnology, Madrid, Spain. All experiments were carried out according to Spanish law for animal welfare and with ethical permission of local authorities. Mating was performed overnight and noon of the day of plug detection was considered as embryonic day (ED) 0.5. Animals were compared to littermates. Genotyping of animals was performed as described before [33] [34].

Total RNA from placentas was isolated using the RNeasy Plus Universal Kit (Qiagen) and reversed transcribed using the iScript Reverse Transcription Supermix (Biorad) according the manufacturer's instructions. Gene Expression was determined using SYBR Green based quantitative PCR in 5 µl total reactions containing 300 nM sequence specific primers, 10 ng cDNA and 2.5 µl LuminoCt SYBR Green qPCR ReadyMix (Sigma). PCR runs were performed on a CFX384 instrument (Biorad) using a 2-step cycle program (denaturation, 95°C for 3 sec; annealing/elongation for 15 sec at 60°C). Data were analyzed using the CFX-Manager software package (Biorad) based on the relative delta-delta Ct expression method. The following primers were used for amplification: DREAM_fwd: CAC CTA TGC ACA CTT CCT CTT CA, DREAM_rev: ACC ACA AAG TCC TCA AAG TGG; Gcm-1_fwd: TGA TCA TGC TCG CCT TTG G, Gcm-1_rev: TGA AGC TTA TTC CCT GCC GA; HPRT-1_fwd: TCT TTG CTG ACC TGC TGG ATT; HPRT-1_rev: TAT GTC CCC CGT TGA CTG ATC; TBP_fwd: CGG ACA ACT GCG TTG ATT TTC; TBP_rev: AGA CCA ACT TCT GCA CAA CTC.

The albumin/creatinin ratio in urine samples is a marker for proper kidney function. Elevated levels indicate potential kidney dysfunctions. Urine samples of pregnant animals were collected at the time of dissection, snap frozen and stored at −80°C. The presence of albumin was tested using the Albuwell M ELISA in combination with a creatinin ELISA (Exocell) for correction for urine dilution. A 10 µl sample was processed according to instructions.

### Immuno-precipitation and Western blot

Placental samples from sPE and aged matched control patients (see section Tissue Collection and Table 1) were crushed under liquid nitrogen and stored at −70°C. Tissue powder was homogenized in lysis buffer [Hepes pH 7.9, 20 mM, NaCl 140 mM; EDTA 1 mM; Triton×100 1%] supplemented with proteases (complete Mini, Roche) and phosphatase (Calibiochem) inhibitor cocktails, and with N-ethylmaleimide to prevent desumoylation. Lysates were cleared by centrifugation at 12,000×g at 4°C and precleared with protein G Sepharose beads (GE Healthcare). Rabbit polyclonal DREAM antibody 1014 [34] was used for immuno-precipitation overnight at 4°C. Antibody bound protein was captured for 2 hrs at 4°C and beads were washed 5 times with lysis buffer. Protein was eluted from the beads with SDS sample buffer. Western blot was probed with a mouse anti-SUMO1 antibody (Zymed). Blots of short exposure time with signal in the linear range were scanned and quantified using QuantityOne (BioRad) and the band intensity related to input (10% of sample after incubation with anti-DREAM antibody).

### Statistical analysis

All experiments were performed in at least triplicates. Data are presented as mean with errors expressed in S.E.M. Unpaired t-test and the Mann-Whitney rank-sum tests were used to test for significance between treatment and the controls. One-way ANOVA and the Tukey's post hoc test were used to compare multiple treatment groups. Data were normalized within culture relative to the negative treatments. Intensity of DREAM staining in TMA was analyzed using the *x* ^2^ and 2-tailed Fisher's exact probability test. All statistical calculations were performed using GraphPad Prism 5.0 software and p-values≤0.05 were considered significant. For the immuo-precipitation experiments the data were analyzed using one-tailed paired t-test.

## Results

### DREAM expression pattern in the human placenta during pregnancy

**Figure 2:**
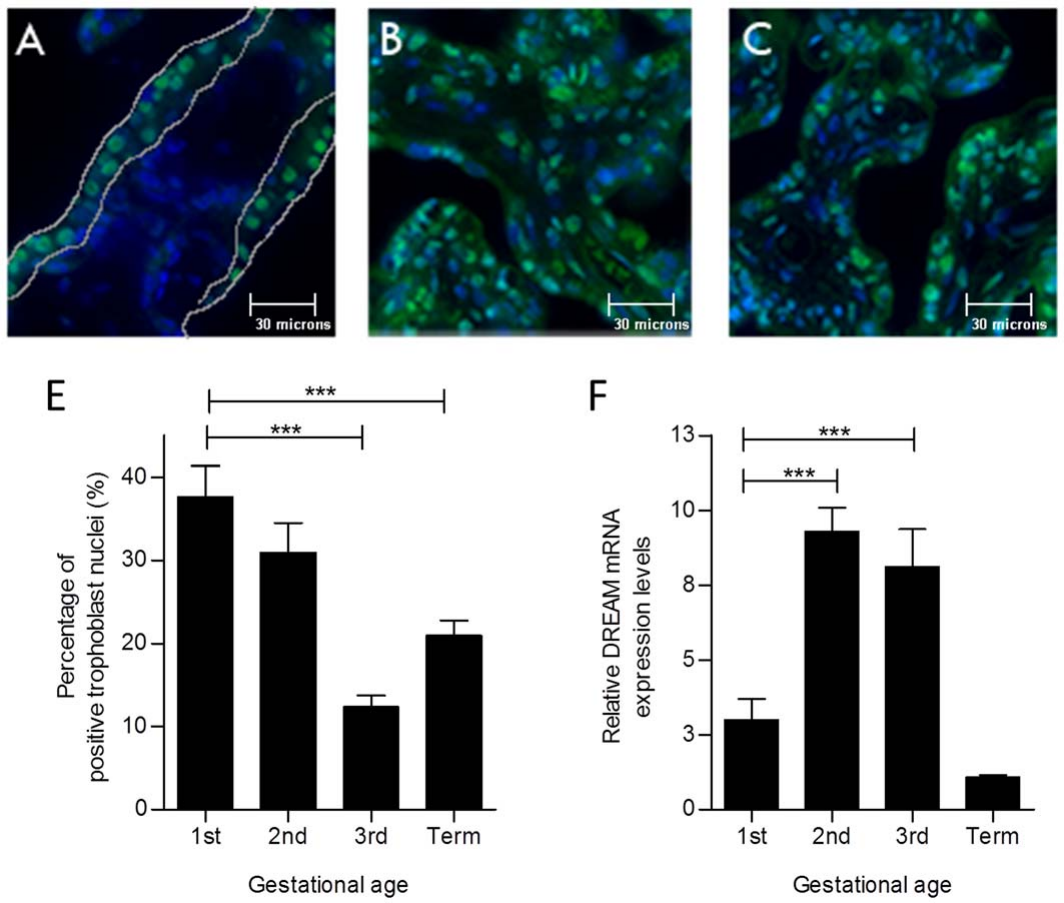
DREAM expression in human placenta of first, second and third trimester. Expression pattern of DREAM was examined using immunofluorescence in 8 week (**A**), 16 week (**B**) and 38 week (**C**) placentas. DREAM is stained green and nuclei are stained blue (DAPI). Each image is a representative of 50 fields examined (original magnification, ×400). (**D**) Quantitative analysis of nuclear expression of DREAM in the trophoblast layer. Number of DREAM-positive nuclei is expressed as a percentage of the total number of nuclei in the trophoblast layer. Nuclear expression decreases with advancing gestation. (E) Total placental DREAM mRNA expression was monitored using qRT-PCR. Data are presented as mean+\−SEM from 3–4 placentas per group (D) and 6–11 placentas per group (E). *** p<0.001.

The distribution of DREAM protein in human placenta was localized by immuno-staining in first, second and third trimester tissue sections of placental villi. DREAM immuno-fluorescence was predominantly localized to nuclei of VCT cells in the first trimester, while a subset of SYN nuclei were also positive (Figure 2A). In the second trimester, DREAM immune-staining was detected in nuclei as well as the cytoplasm of both trophoblast and stromal cells (Figure 2B). In the third trimester, expression persisted in the trophoblast layer and stroma but was weaker and predominantly cytoplasmic (Figure 2C).

We quantified the nuclear DREAM expression within villous trophoblast layer throughout gestation (Figure 2D). According to our observations, the percentage of DREAM immuno-positive villous trophoblast nuclei declined significantly from 37.6±3.7% in the first trimester to 12.4±1.4% in the third trimester (p<0.001). Stromal DREAM expression was not quantified but our immuno-fluorescence staining showed marked up regulation of DREAM expression in the second and third trimesters. Thus, the overall placental DREAM mRNA expression was the highest in the second and third trimester samples (Figure 2E).

### DREAM interacts with the human GCM1 promoter

*In silico* analysis of the 5 kb region encompassing the human GCM1 promoter revealed numerous downstream response element (DRE) core sequences (GTCA) – a known binding site of DREAM transcriptional repressor. Of note, some DRE sites in the GCM1 promoter appear in clusters/tandems of two or three sites (Figure 1), a disposition that has been associated with an enhanced affinity to bind multimeric DREAM complexes and has been reported in the promoters of some DREAM target gens e.g. NCX3 [23].

**Figure 3:**
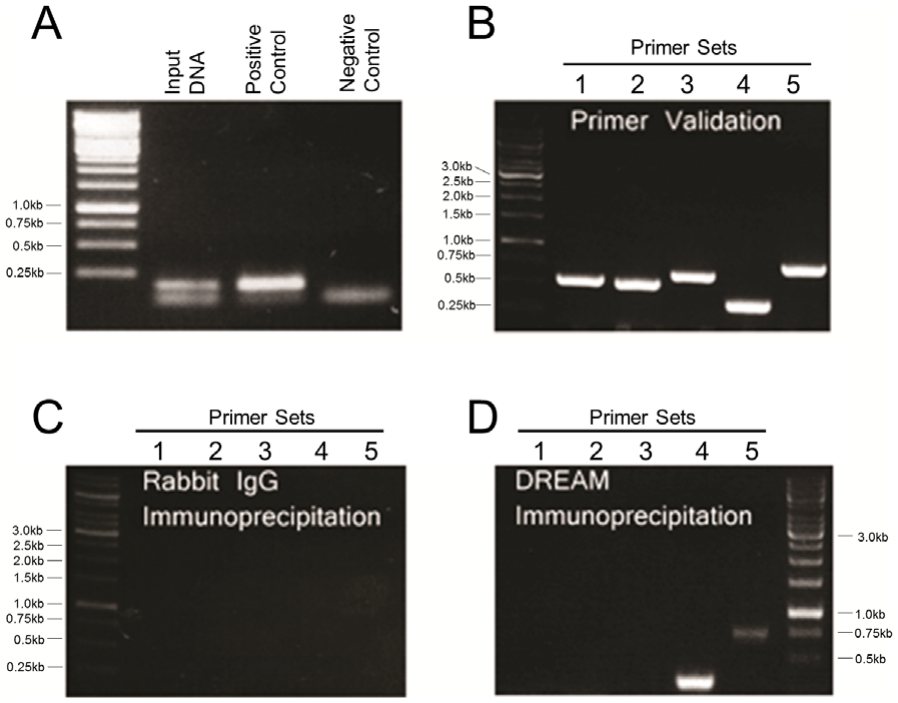
ChIP analysis of the GCM1 human promoter. (**A**) ChIP assay was validated with positive and negative controls as indicated. (**B**) Five designed primer sets were validated using a BAC clone containing the human GCM1 promoter as DNA template. (**C**) As a negative control rabbit IgG-immuno-precipitated DNA was subjected to PCR amplification with the five primer sets. (**D**) DREAM-immuno-precipitated fraction was subjected to PCR amplification with the five sets of primers. Amplification was observed with primer sets 4 and 5.

To investigate DREAM protein and GCM1 promoter interactions we employed chromatin immuno-precipitation (ChIP) assay using BeWo cells, a cell line with regulated GCM1 expression [11]. DREAM immuno-precipitated fractions were reverse cross-linked and subjected to PCR amplification with five different sets of PCR primers (Table 3) designed to amplify regions in the human GCM1 promoter. The strongest amplification was observed with primer set number 4 (Figure 3D and Figure 1), suggesting DREAM binding within this region of the promoter. In addition, a weak amplification signal was obtained with primer set 5 (Figure 3D and Figure 1). As a control, similar results were obtained in experiments performed when His-tagged DREAM was over-expressed in BeWo cells and immuno-precipitated with anti-HIS antibody (unpublished observation).

To further investigate the interaction between DREAM protein and GCM1 promoter we performed electrophoretic mobility shift (EMSA). Nuclear extracts from BeWo cells over-expressing His-tag labeled DREAM or treated with siRNA to silence endogenous DREAM were used together with labeled amplicons of primer sets 1 to 5. Over-expression of His-tagged DREAM or treatment with siRNA increased 5000-fold or reduced by 70%, respectively, DREAM mRNA levels in BeWo cells (see later in Figure 5). Importantly, binding of DREAM to a 343 bp probe corresponding to amplicon 4 of the GCM1 promoter (Figure 1) generated a retarded band (Figure S1A, lane 4) that was weaker when a nuclear extract from DREAM-silenced BeWo cells were used (Figure S1B, lane 4). The retarded band was competed with unlabeled probe. No binding was obtained with amplicon 5 (Figure S1).

**Figure 4:**
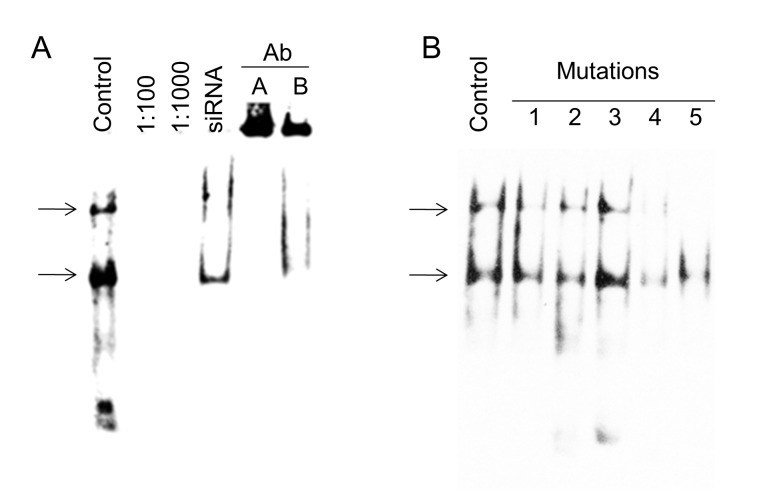
EMSA of 43 bp sequence from the GCM1 promoter. (**A**) Binding to a 43 bp 3′ end biotin-labeled probe was tested on nuclear fraction from DREAM-overexpressing cells (control) produced two bandsdenoted with arrows)s. 1∶100 and 1∶1000 competition with unlabeled probe was performed. The probe in combination with the nuclear extract isolated from DREAM siRNA-treated cells resulted in decreased abundance of both bands. Successful super-shift analysis with anti-Histidine tag Ab(A) and anti-DREAM Ab(B) was performed. (**B**) Mutation analysis of the DRE sites in the 43 bp sequence. Mutated probes were tested using nuclear extracts from DREAM-over-expressing BeWo cells. Mutations 1–3 did not alter the binding DREAM to the probe. The binding of mutated probes 4 and 5 to nuclear extract from DREAM overexpressing BeWo cells resulted in reduced signal.

Thus, ChIP and EMSA both point to a direct interaction between DREAM protein and the GCM1 promoter within a 343 bp area of region 4. Careful examination of this region uncovered 3 core DRE binding sites located within a 43 bp area of the GCM1 promoter (Figure 1). A short probe corresponding to this 43 bp sequence generated two bands in EMSA (Figure 4) both of which were outcompeted using unlabeled probes (1∶100 and 1∶1000). As expected, much weaker signals were observed in nuclear extracts from DREAM silenced cells and both bands were “supershifted”utilizing anti-His tag and anti-DREAM antibodies (Figure 4A).

To examine the affinity of DREAM protein binding to the individual 3 DRE sites, various mutations were introduced (Table 4). One nucleotide mutations in individual DRE sites (mutation 1–3) did not affect the binding of DREAM (Figure 4B). Reduced affinity, however, was observed with probes containing multiple mutation sites (Figure 4B). In conclusion, EMSA confirmed binding of DREAM to DRE sites within a 43 bp sequence in the human GCM1 promoter. Our results however, limit our ability to claim specificity of the GCM1-DRE binding as the “supershift” may be a result of non-specific interaction between antibody and protein-DNA complexes.

### DREAM regulates GCM1 transcription in a Ca^2+^-regulated manner

**Figure 5:**
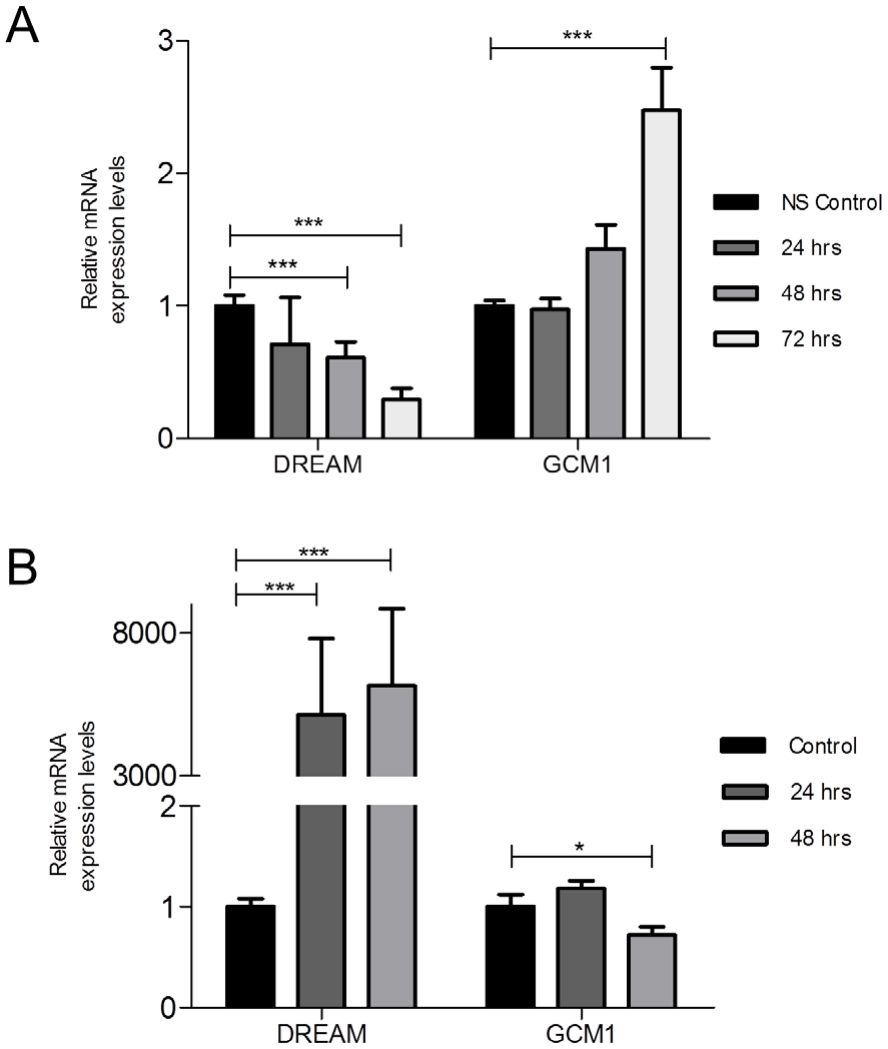
Changes in DREAM expression modified GCM1 levels in BeWo cells. (**A**) DREAM silencing of BeWo cells increased GCM1 mRNA levels. (**B**) DREAM over-expression in BeWo cells reduced GCM1 mRNA levels. Data presented as mean+\−SEM from 4 independent experiments with each value determined in triplicate. Each treated time point was compared to an equivalent non-silenced time point control. All non-silenced control time points are set as 1. *** p<0.001, * p<0.05. NS, non-silenced.

Since DREAM binds to the GCM1 promoter, we next investigated the functional consequence of GCM1 gene expression. Reduction of DREAM mRNA levels by 70% after treatment of BeWo cells with 50 nM DREAM siRNA resulted in 2.5 fold increase in GCM1 mRNA levels as compared to non-silenced controls (Figure 5A). Consequently, increased DREAM mRNA levels after over-expression in BeWo cells (5,130±2,520 vs 1.0±0.09, p<0.001 in non-transfected cells) reduced GCM1 mRNA levels at 48 hrs post transfection as compared to controls (0.72±0.08 vs 1.0±0.1, p<0.05) (Figure 5B). The relatively small decrease in GCM1 levels, resulting from 5000-fold increase in DREAM, might reflect the already relatively high DREAM expression observed in the first trimester trophoblast cells.

**Figure 6:**
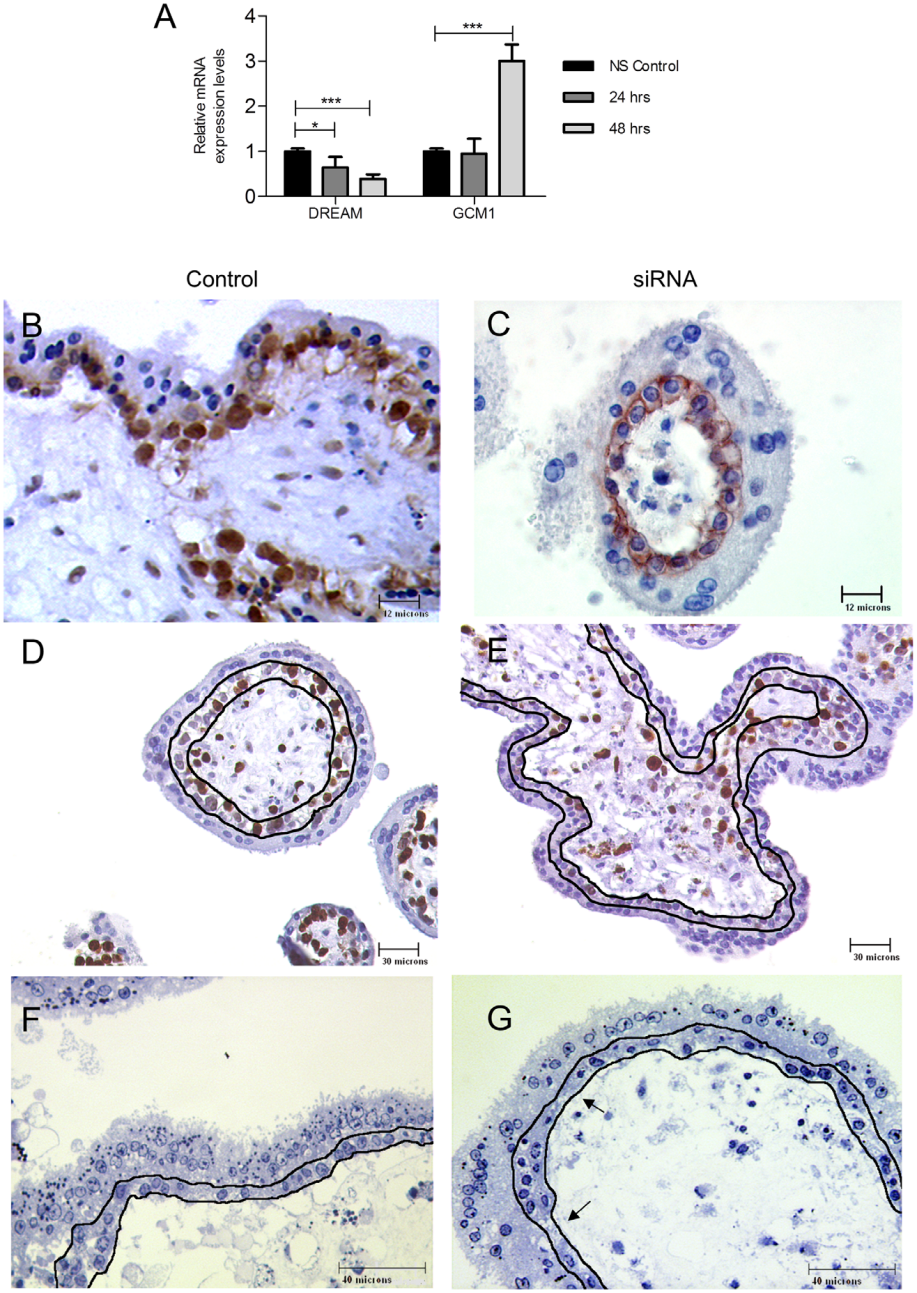
DREAM silencing in first trimester floating placental villous explants. (**A**) Increased GCM1 expression in placental explants after siRNA-mediated DREAM silencing. Results are mean+\−SEM from 5 placentas per group with each value determined in triplicate. Each treated time point was compared to an equivalent non-silenced time point control. All non-silenced control time points are set as 1. Histological assessment of DREAM siRNA-treated first trimester floating villous explants revealed reduced expression of DREAM (**B, C**) and Ki-67 (**D, E**) in siRNA-treated explants as compared to non-silenced control explants following 2 days of culture. Semi-thin histology of non-silenced control (**F**) and DREAM siRNA-treated (**G**) explants with the cytotrophoblast layer delineated. Following siRNA treatment, villous cytotrophoblast layer became dis-continuous (arrows) and showed nuclear condensation in some areas (*). Each field is a representative of 50 samples examined (original magnification; B, C = 1000×; D, E, F, G = 400×). *** p<0.001, * p<0.05. NS, non-silenced.

Studies were also conducted in first trimester floating villous placental explant model. Placental explants (8–12 week) were cultured in the presence of 100 nM DREAM siRNA or 100 nM non-silencing control for up to 48 hrs. Treatment of explants for 2 days with DREAM siRNA reduced DREAM mRNA to 0.39±0.09, p<0.001 fold of control and resulted in 3.1±0.36, p<0.001 fold GCM1 mRNA up-regulation at the same time point as compared to non-silenced controls (1.0±0.08) (Figure 6A). These observations could not be validated with western blotting for DREAM as the available antibodies were unreliable. DREAM monomer, dimmer and tetramer forms as well as modifications such as sumoylation further obscured the quantitative analysis.

DREAM siRNA-treated explants demonstrated much weaker immunopositivity to DREAM antibody as compared to non-silencing explants. In siRNA treated explants DREAM staining was predominantly localized to the cytoplasm and membranes unlike the control counterpart where DREAM nuclear expression was observed (Figure 6B–C). DREAM siRNA treated explants also showed a markedly reduced staining for Ki-67 proliferation marker within the trophoblast layer, suggesting reduced VCT rate of proliferation (Figure 6 D–E).

Trophoblast morphology of DREAM siRNA treated explants was further examined using toluidine blue stained, semi-thin sections. Following two days of culture, non-silenced control explants retained a continuous single layer of VCT cells underneath an intact syncytial layer (Figure 6F). In DREAM siRNA treated explants, the VCT layer became dis-continuous suggesting cell depletion. Furthermore, some of the VCT showed nuclear condensation and the overlying syncytial layer contained a single to double layer of new, large and pale euchromatic nuclei (Figure 6G). These observations suggest depletion of VCT progenitors due to acceleration of SYN fusion by GCM1 induction.

DREAM has been reported to play a role in the inhibition of apoptosis through negative regulation of the pro-apoptotic protein gene hara-kiri (Hrk) (via interactions with Bcl-2 and Bcl-X(L)) in hematopoietic progenitors [35]. Thus the Hrk mRNA levels were monitored in the DREAM siRNA treated explants with two objectives; first, to confirm DREAM's functional repression following siRNA treatment, and second to assess the rate of apoptosis in these explants. Interestingly, Hrk mRNA levels could only be detected in DREAM siRNA treated explants following 48 hr of treatment (Figure S2), confirming Hrk as a DREAM target in these cells and further, suggesting a role for DREAM in controlling apoptosis in human placenta.

**Figure 7:**
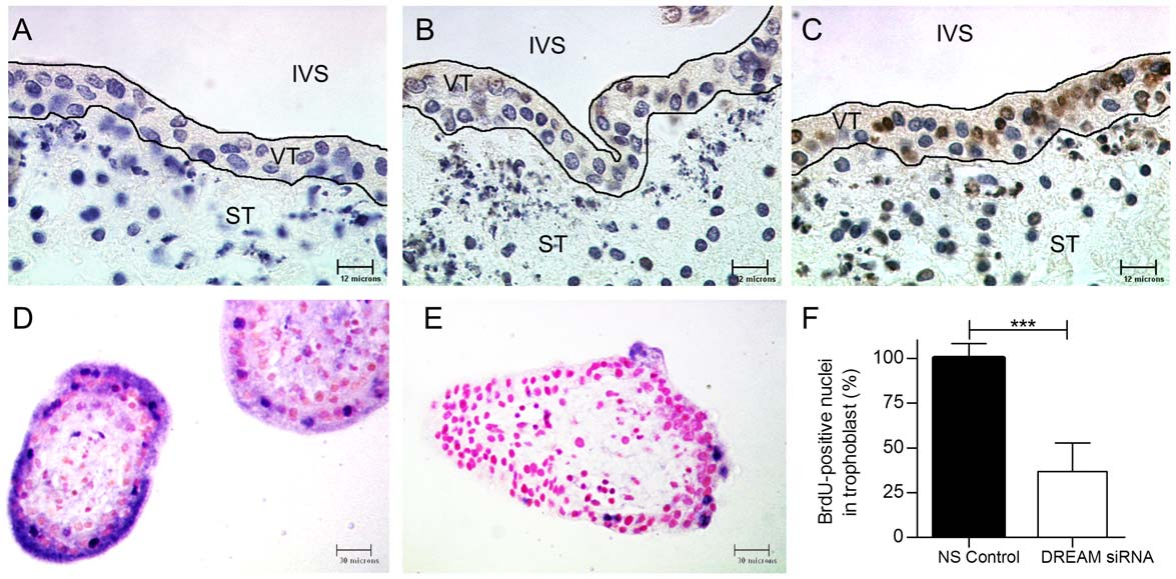
Apoptosis and proliferation assays in DREAM siRNA-treated explants. TUNNEL staining was performed in (**A**) t = 0 hr control, (**B**) 100 nM non-silenced control and (**C**) 100 nM DREAM siRNA-treated explants cultured for 48 hrs. Pictures are representatives of 30 images from 5 independent experiments. Positive nuclear staining was observed only in DREAM siRNA-treated explants. (**D–E**) BrdU staining in non-silenced control (**D**) as compared to DREAM siRNA-treated (**E**) explants. BrdU incorporation was visualized with anti-BrdU immunohistochemistry. BrdU (10 µM) was added to the culture media of the explants for the duration of each of the experiments. (**F**) Proliferation was measured as the number of BrdU-positive trophoblast nuclei per total number of trophoblast nuclei and normalized to the control. The control was set at a 100% Results are mean+\−SEM from 5 placentas per group with each value determined in triplicate. *** p<0.001. IVS, inter-villous space; VT, villous trophoblast; ST, stroma. Magnification A–C 1000×, D–E 400×.

The rate of trophoblast proliferation and apoptosis in DREAM siRNA treated explants was monitored with BrdU incorporation and TUNEL staining studies respectively. No TUNEL positive nuclei were detected in the time = 0 control or in the non-silencing control, while DREAM siRNA treated cells displayed strong TUNEL staining within the trophoblast layer (Figure 7A–C). Conversely, 2 days treatment of explants with 100 nM DREAM siRNA resulted in significant reduction in the rate of VCT proliferation as compared to non-silenced control (37±16% vs 100±8%, p<0.001) (Figure 7F).

**Figure 8:**
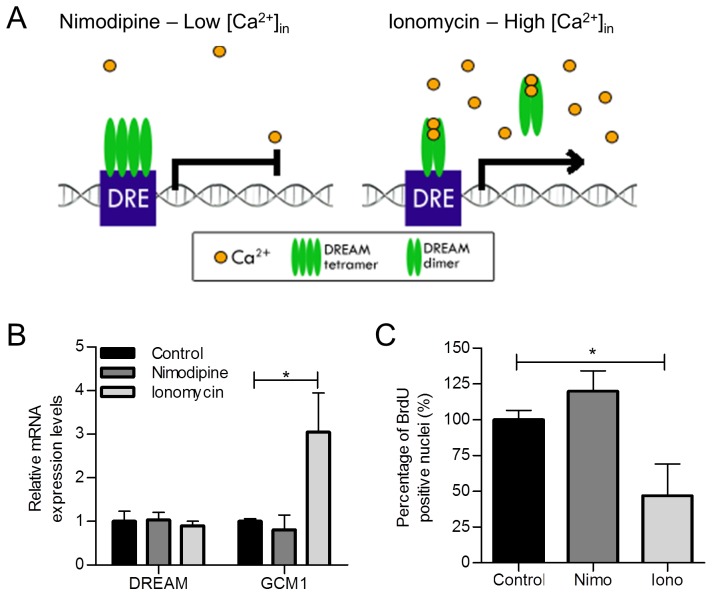
Nimodipine and ionomycin treatment of first trimester floating villous explants. (**A**) Diagrammatic representation of DREAM and DRE interactions under altered intracellular calcium concentrations. (**B**) Reduced GCM1 mRNA levels after ionomycin treatment. Data presented as mean ± SEM from 5 independent experiments. (**C**) BrdU incorporation was used to measure altered cytotrophoblast rate of proliferation in nimodipine and ionomycin-treated first trimester placental explants. Proliferation was measured as the number of BrdU-positive trophoblast nuclei per total number of trophoblast nuclei and normalized to their respective controls. The control was set at a 100% * p<0.05. DRE, downstream regulatory element; Nimo, nimodipine; Iono, ionomycin.

Finally, since DREAM is a calcium-dependent transcriptional repressor, we studied changes in GCM1 expression and VCT proliferation as a result of changing intracellular calcium concentrations. Exposure of first trimester placental explants to the calcium ionophore ionomycin resulted in a significant 3-fold up-regulation of GCM1 mRNA levels in the explants (Figure 8A) and a significant reduction (over 50%, p<0.05) in the rate of VCT proliferation as compared to control explants (Figure 8B) without an effect on DREAM mRNA levels (Figure 8A). Nimodipine stimulation of first trimester explants had no significant effect on the rate of VCT proliferation as compared to its control (Figure 8B).

### DREAM expression and sumolyation are increased in sPE placental villi

**Figure 9:**
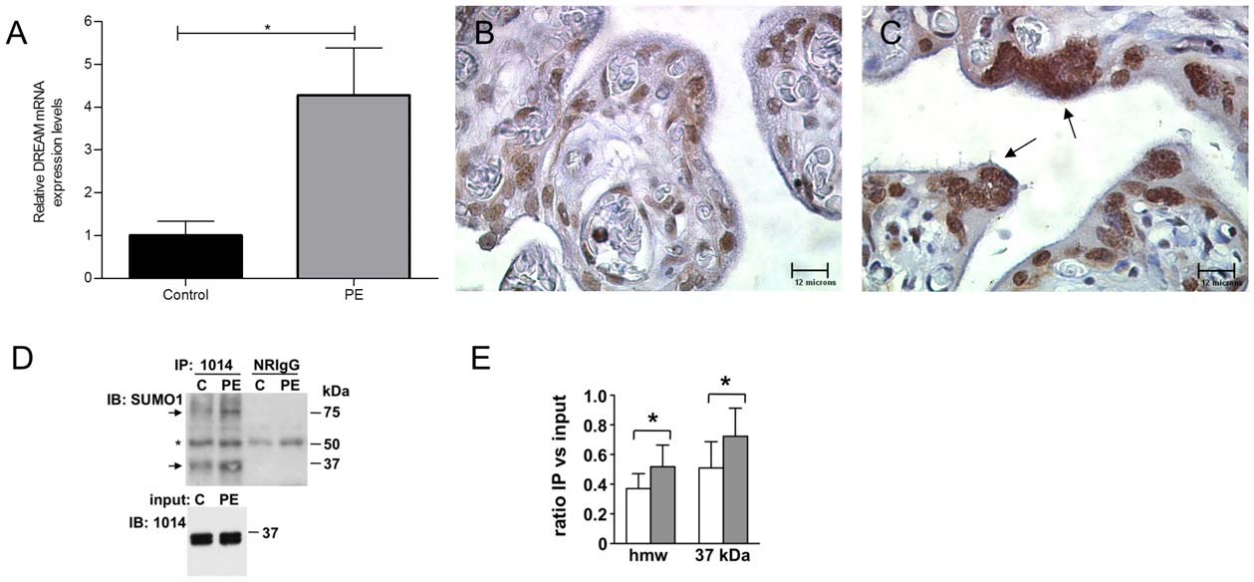
Increased DREAM expression in severe preeclamptic (sPE) placenta and immuno-precipitation analysis of sumoylated DREAM. (**A**) qPCR analysis of DREAM levels. Results are mean+\−SEM from 6 placentas per group. DREAM immunohistochemistry was performed on the RCWIH BioBank TMA #1 array containing 8 sPE samples and 6 age-matched controls. Representative images from control (**B**) and sPE (**C**) are shown. Strong nuclear immunostaining was observed in syncytial knots of the sPE placentas (arrows). (**D**) DREAM IP with rabbit polyclonal antibody 1014 or normal rabbit IgG was subjected to immunoblot analysis with anti-SUMO1 Ab. Arrows represent specific sumoylated DREAM bands. Asterisk labels a non-specific immuno-reactive band. Input (10%) after incubation with the DREAM Ab. One representative pair of control (C) and sPE placental tissue is shown. (E) Graph representing the ratio of IP bands intensity versus input from 6 controls and 6 sPE samples. * p<0.05. hmw, high molecular weight; PE, preeclampsia. Magnification B–C 1000×.

sPE is associated with reduced levels of GCM1 [16]. Since we demonstrated a negative regulatory role of DREAM for GCM1, we determined the expression pattern of DREAM in this placental pathology. In comparison with age-matched controls, sPE placentas exhibited increased DREAM mRNA levels (4.28±1.1 vs 1±0.33, p<0.05) (Figure 9A). To confirm these results at the protein level, tissue arrays containing sPE placentas and aged-matched controls were immunoassayed for DREAM. Diffused staining in the entire tissue with individual stronger nuclear expression in the trophoblast layer was observed in control placentas. In line with our qRT-PCR results, sPE samples revealed strong nuclear expression of DREAM in the trophoblast layer; especially in syncytial knots, a key histo-pathologic feature that is characteristic of sPE (Figure 9B). Blind scoring test of strong vs weak DREAM staining in sPE and age-matched control samples revealed significantly (p<0.001) elevated levels of strong immunopositivity in sPE placentas. In sPE placentas 24 of 32 cores (4 cores from 8 placentas) showed strong DREAM immunopositivity as opposed to only 5 of 24 in age-matched controls (4 cores from 6 placentas).

Since nuclear localization has been associated with sumoylation of DREAM, we analyzed this post-translational modification in total extracts from sPE placentas. Immuno-precipitation with a DREAM antibody followed by Western blot with a SUMO-1 antibody revealed a significant increase in monosumoylated DREAM protein in sPE vs control placental tissue (0.72±0.19 vs 0.51±0.18, p<0.05). Also, a similar increase in sumoylated multimeric-DREAM (0.52±0.15 vs 0.37±0.10, p<0.05) was detected in sPE vs control placental tissue (Figure 9D).

### Analysis of DREAM –deficient and DREAM overexpressing mice

DREAM expression was not altered in the placentas of the DREAM overexpressing mice indicating that the transgene is not active in the placental tissues (Figure S3G). No significant differences in embryonic or placental weights were observed between wild type, heterozygous and knock out littermates at ED 13.5 or ED 18.5 (Figure S3A–D).

DREAM expression was reduced in heterozygous DREAM-deficient placentas compared to wild type placenta and absent in the knockout placentas (unpublished observations). Gcm-1 mRNA levels, however were not changed in placentas of heterozygous or knockout DREAM-deficient mice compared to wild type littermates at ED 13.5 (Figure S3F)

## Discussion

This study defines the expression and function of the calcium dependent transcriptional regulator DREAM in the human placenta. Specifically, we have validated the participation of DREAM in controlling GCM1-directed differentiation of SYN in human placental villi. Using a loss of function approach in two culture models, the chorio-carcinoma cell line BeWo and first trimester placental explants, we were able to demonstrate that DREAM negatively regulates transcription of GCM1 and thus restricts villous trophoblast turnover. Up-regulation of DREAM expression in the BeWo cell model resulted in inhibition of GCM1 mRNA expression. ChIP and EMSA results suggest the specific and cooperative binding of DREAM to DRE sites within a 43 bp sequence of the human GCM1 promoter.

The ability to sustain SYN fusion events throughout human pregnancy, and thus the integrity of the outer SYN layer relies on a delicate balance of proliferation, differentiation and syncytial fusion of a subset of VCT cells [36]. The placental insufficiency disorder of sPE is often associated with IUGR; in this condition, analysis of pathologic placental villi demonstrates depletion of proliferating VCT accompanied by defects in the outer SYN layer [15]. These histo-pathologic observations are accompanied by reduced expression of GCM1 [16] and syncytin [17]. Previously we demonstrated *in vitro* that GCM1 controls the balance between proliferation and differentiation of both pathways (villous and extravillous) of human trophoblast development [11].

In keeping with the transcriptional repressor activity of DREAM in the nucleus, we observed strong nuclear expression in the villous trophoblast layer during early gestation when rapid VCT proliferation is needed to expand the villous trees and transform the uteroplacental arteries. As development proceeds, the cell-specific requirements change. Thus, during the second and third trimester we observed much greater DREAM expression in mesenchymal cells, as the villous trees transform from immature to mature intermediate villi [1]. Since GCM1 is not found in placental stroma, DREAM may have different downstream targets at these later stages.

Functional studies in the BeWo cell culture model indicated that DREAM is a negative regulator of GCM1 expression. Additionally, the experiments performed in the more physiologic human floating villous explant model demonstrated that alterations in DREAM expression impact on GCM1 mRNA levels, VCT proliferation and SYN differentiation and apoptosis. In first trimester floating villous explants, reduced DREAM increased GCM1 mRNA expression and inhibited VCT proliferation. The short culture period did not result in degeneration of the overlying SYN assessed by semi-thin sections, though induction of apoptosis (up-regulation of the pro-apoptotic protein Hrk and positive TUNEL staining in the trophoblast layer) was seen. Therefore, we speculate that a sustained (>48 hrs) down-regulation of DREAM expression is required to induce degeneration of the SYN layer *in-vitro*.

*In silico* analysis of the human GCM1 promoter identified potential DREAM binding sites (DRE). Our ChIP and EMSA studies of the GCM1 promoter disclosed a 43 bp area containing 3 DRE sites exhibiting affinity for DREAM protein. Interaction of DREAM with the promoter regions of a number of genes including, prodynorphin [19], NCX3 [23], cytokines [34] and calcitonin [37] was reported previously. Data presented in this study are novel in that we have identified, by mutation analysis, binding of DREAM to three tandem repeats of the DRE sequences within the 43 bp area located in the human GCM1 promoter.

To attest that DREAM is a physiologic regulator of GCM1 we turned to two mouse models. In DREAM knockout mouse we found: i) no differences in the placental GCM1 expression levels, ii) no changes in the placental development (weight, morphology), and iii) no changes in embryonic weights (Figure S3), possibly due to gene compensation by the other members of the DREAM/KChIP family of proteins [34], [38]. Conversely, given our findings of over-expression of DREAM in sPE placentas, we focused on a DREAM over-expressing mouse model [23] [28], hypothesizing that this strain will display pre-eclamptic-like signs. Although this transgenic mouse over expresses a constitutively active human DREAM mutant under the specific promoter (CaMK IIα) that directs expression mainly in the brain, mouse lines with ubiquitous expression outside the central nervous system have been characterized [34]. Unfortunately, transgene expression was not observed in the placenta/trophoblast linage in these ubiquitous lines and pup and placental weights, maternal blood pressure, proteinuria levels, GCM1, syncytin, sFLT expression levels and kidney morphology were not different from values from wild type littermates (Figure S3). Hence, the only currently-existing DREAM over-expressing mouse is not an appropriate model to study murine placental development.

Our observations in first trimester floating human placental explant model suggest calcium-regulated DREAM activity such that low availability of calcium will increase the biologic activity of DREAM, thereby repressing GCM1. Stimulation of explants with ionomycin resulted in significant up-regulation of GCM1 mRNA levels and inhibition of VCT proliferation. These observations suggest that the up-regulation of GCM1 expression over gestation [10] and decreased rate of VCT proliferation in later gestation may be linked to reduced DREAM activity via increased intracellular calcium levels. Various calcium mediated signal transduction pathways are implicated in placentation and placental function (for a review see [39]). Specifically, previous report has linked elevated levels of intracellular calcium with the increased rate of VCT differentiation [40]; however this is the first report to specifically identify the possible molecular mechanism of calcium-regulated trophoblast turnover.

Interestingly, our investigation of DREAM expression in sPE placentas revealed significant up-regulation of this transcriptional repressor. This observation correlates with the previous report demonstrating placental GCM1 down regulation in this maternal disease [16] and our *in vitro* data where down regulation of GCM1 in villi recapitulated several histologic features of sPE [11]. Furthermore, we found high levels of DREAM protein in post-mitotic syncytial knots in placentas from sPE pregnancies (Figure 9). Since the syncytial layer retains transcriptional activity [41] we explored the hypothesis that DREAM over-expression could occur focally in these knots following syncytial fusion. From our *in silico* analysis, we speculated that DREAM could be de-repressed in sPE via hypo-methylation of critical sites in the promoter region, though we observed no such changes (File S1). An alternative explanation is that DREAM persists in a functional state for longer than under normal circumstances. Previous reports indicate that DREAM retains its active transcriptional repression function following caspase-3 cleavage [42], an event that occurs in SYN as it differentiates along the apoptosis pathway [7], and is resistant to oxidative damage [43]. Finally, we explored sumoylation as a potential mechanism of retention of DREAM and found elevated levels of sumoylated protein in sPE as compared to age-matched controls (Figure 9D). Indeed, the increased level of sumoylated DREAM could account for its increased nuclear presence and a greater repressor capability. Whether sumoylation and nuclear retention is associated with a reduced turnover of the DREAM protein has not been currently demonstrated.

A final aspect important to explaining GCM1 repression in this pathology is the local availability of calcium. Systematic review and meta-analysis of intervention trials of calcium supplementation to prevent pre-eclampsia show a 59% reduction in the risk of this disease in developing countries [30]. Since low calcium availability increases the local actions of DREAM to repress GCM1, at a global level, this observation may have great importance, and our study cast important light on a pathway to mediate better placentation in this setting.

Our findings provide a molecular explanation for the reported decrease in GCM1 and the abnormal VCT, SYN turnover in the sPE placentas. This work makes a significant contribution towards advanced understanding of the regulatory mechanism of this important transcriptional factor in proper placental establishment, development and function.

## Supporting Information

Figure S1 **EMSA on nuclear extracts from DREAM-overexpressing and DREAM-silenced BeWo cells.** Nuclear extract from DREAM-over-expressing BeWo cells was incubated with 3′ end biotin-labeled amplicons from the five primer sets used in ChIP analysis as in Figure 3. (**A**) The strongest signal was observed in lane 4. Binding of each of the labeled probes was competed in turn with its equivalent unlabeled probe (1∶100). Negative control lane contains the nuclear extract but not the probe. (**B**) The procedure was repeated on nuclear extracts from DREAM-silenced BeWo cells resulting in a much weaker signal. Positive control lane (Panel B) contains probe 4 and nuclear extract from untreated BeWo cells.
(TIF)

Figure S2 **Hrk mRNA levels in DREAM siRNA treated explants.** Hrk qRT-PCR was performed on control (t = 0) explants as well as in DREAM siRNA-treated and non-silencing control explants following 24 and 48 hrs of treatment. Products of the reactions were run on 1.5% agarose gel. Amplification of Hrk mRNA was observed only following 48 hrs of DREAM siRNA treatment. NS = non silencing control, si = DREAM siRNA treatment, M = marker.
(TIF)

Figure S3 **Phenotype analysis of DREAM-deficient (A–F) and DREAM over-expressing (G) mice at gestational days ED13.5 and ED18.5, respectively.** No significant differences in embryonic (A, C) or placental weights (B, D) were observed between wild type (WT), heterozygous (HZ) and knockout (KO) littermates at ED13.5 (A, B) or ED18.5 (C, D) after crossing heterozygous parents. Pregnant animals did not show elevated urine protein levels compared to non-pregnant controls (npc) as indicated by analysis of the albumin/creatinin ratio in urine samples collected at the time of dissection (E). Gcm-1 mRNA levels were not changed in placentas of heterozygous or knockout DREAM-deficient mice compared to wild type littermates at ED13.5 as shown by quantitative real-time PCR (F). In addition, in the transgenic DREAM-over-expressing mouse strain JN1, no ectopic placental expression of DREAM could be detected as revealed by quantitative real-time PCR (G). Data are show as averaged values with standard deviations of a minimum of three litters per gestational day. No statistical differences were detected performing a 1-way ANOVA test followed by Bonferroni post-hoc test in GraphPad Prism 5.0.
(TIF)

File S1 **Supplementary Methods and** **Results** **.** DREAM DNA methylation analysis indicates that this epigenetic mechanism is not responsible for de-repression of DREAM in sPE placentas.
(DOC)
