# Supplementary figures and images for: DREAM Mediated Regulation of GCM1 in the Human Placental Trophoblast
Source: PLoS One. 2013 Jan 3;8(1):e51837. doi: 10.1371/journal.pone.0051837 (PMC3536794; doi:10.1371/journal.pone.0051837)

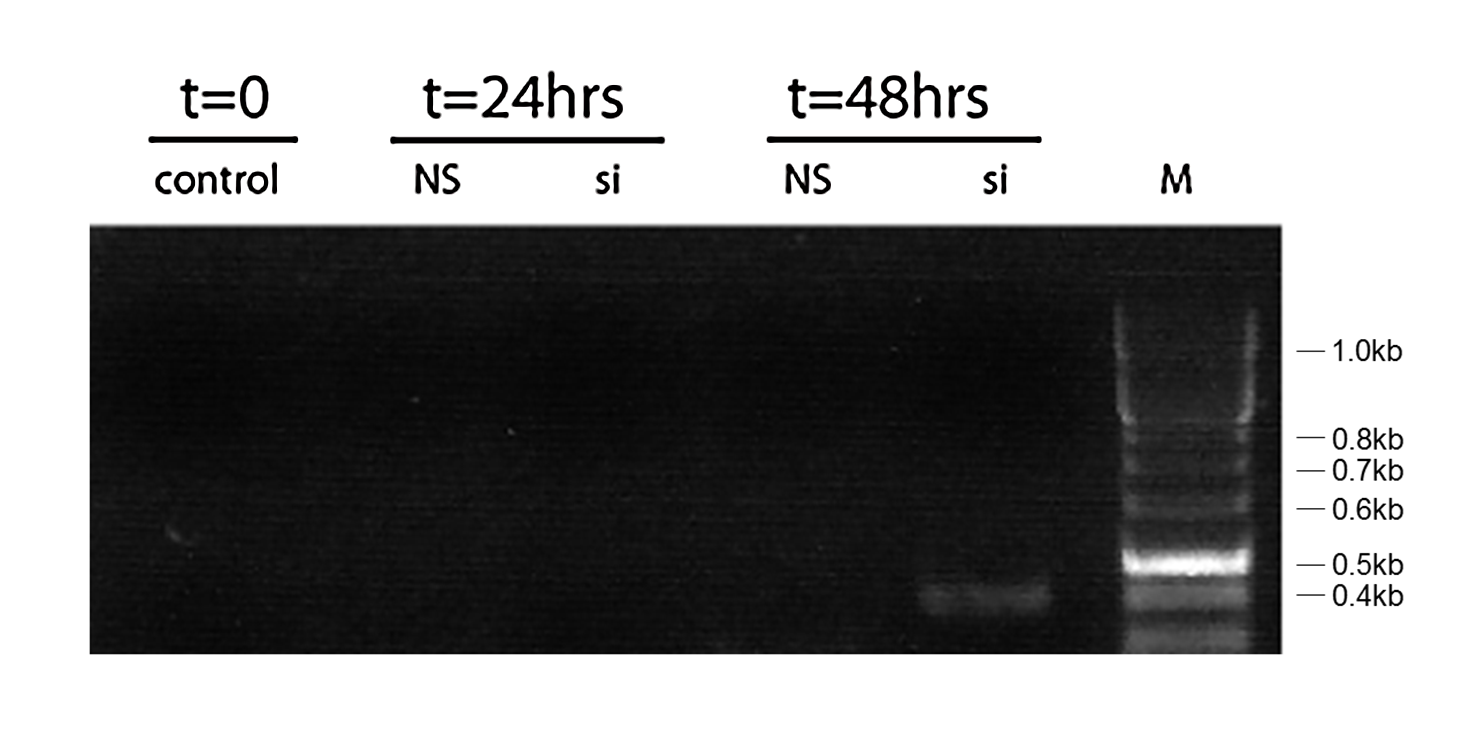

Supplement: Figure S1 — EMSA on nuclear extracts from DREAM-overexpressing and DREAM-silenced BeWo cells. Nuclear extract from DREAM-over-expressing BeWo cells was incubated with 3′ end biotin-labeled amplicons from the five primer sets used in ChIP analysis as in Figure 3. (A) The strongest signal was observed in lane 4. Binding of each of the labeled probes was competed in turn with its equivalent unlabeled probe (1∶100). Negative control lane contains the nuclear extract but not the probe. (B) The procedure was repeated on nuclear extracts from DREAM-silenced BeWo cells resulting in a much weaker signal. Positive control lane (Panel B) contains probe 4 and nuclear extract from untreated BeWo cells. (TIF) [file pone.0051837.s001.tif]

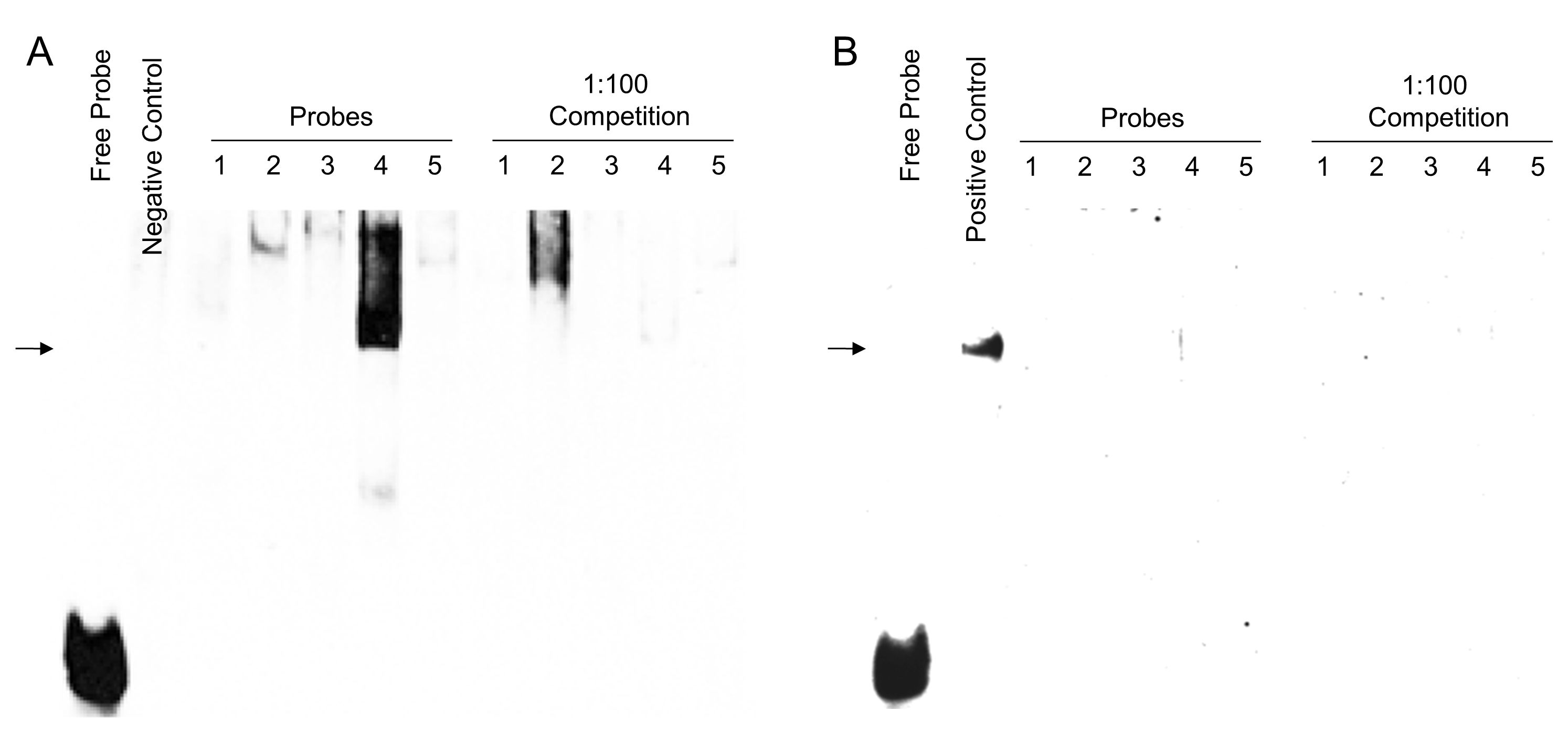

Supplement: Figure S2 — Hrk mRNA levels in DREAM siRNA treated explants. Hrk qRT-PCR was performed on control (t = 0) explants as well as in DREAM siRNA-treated and non-silencing control explants following 24 and 48 hrs of treatment. Products of the reactions were run on 1.5% agarose gel. Amplification of Hrk mRNA was observed only following 48 hrs of DREAM siRNA treatment. NS = non silencing control, si = DREAM siRNA treatment, M = marker. (TIF) [file pone.0051837.s002.tif]

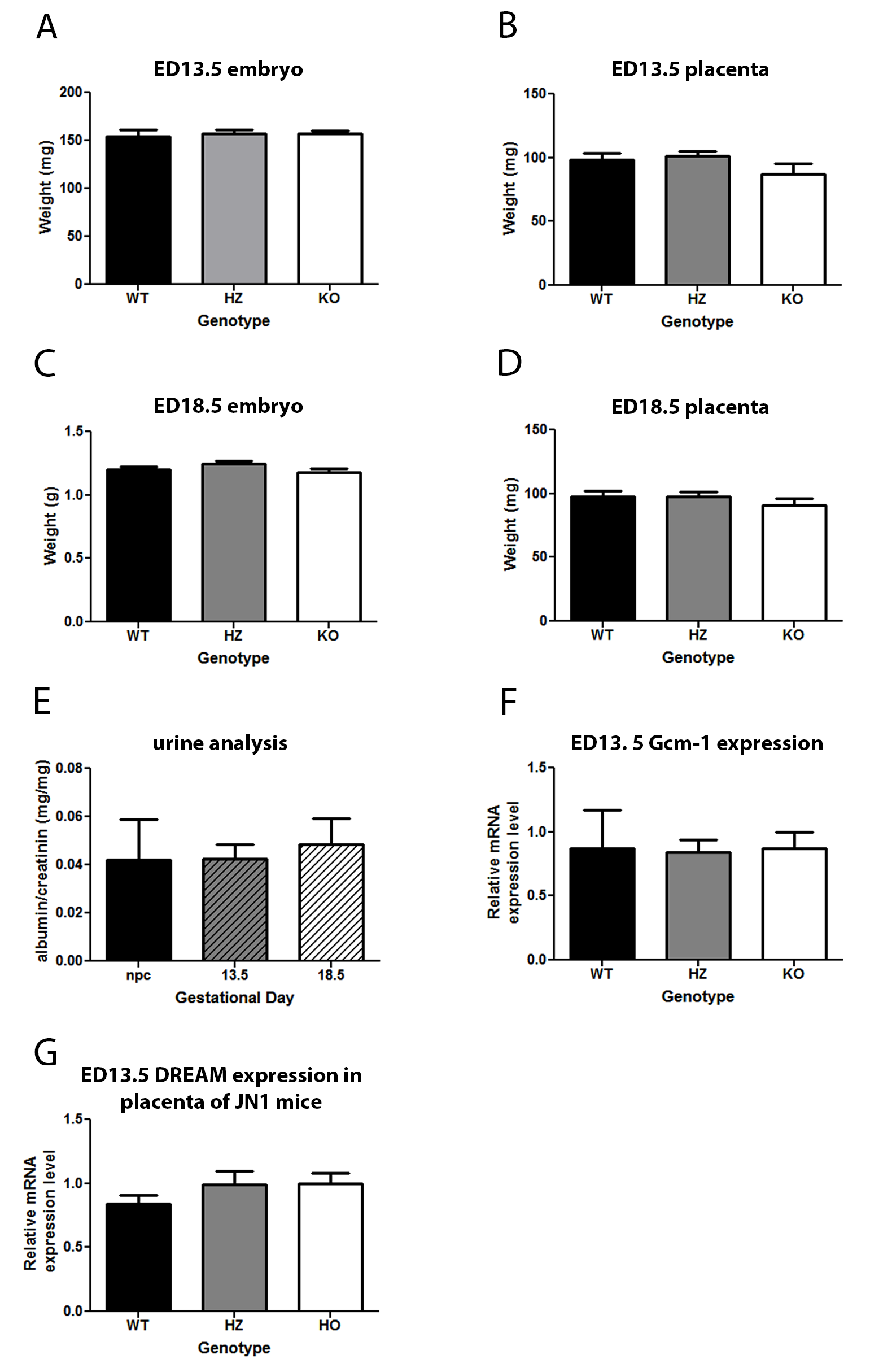

Supplement: Figure S3 — Phenotype analysis of DREAM-deficient (A–F) and DREAM over-expressing (G) mice at gestational days ED13.5 and ED18.5, respectively. No significant differences in embryonic (A, C) or placental weights (B, D) were observed between wild type (WT), heterozygous (HZ) and knockout (KO) littermates at ED13.5 (A, B) or ED18.5 (C, D) after crossing heterozygous parents. Pregnant animals did not show elevated urine protein levels compared to non-pregnant controls (npc) as indicated by analysis of the albumin/creatinin ratio in urine samples collected at the time of dissection (E). Gcm-1 mRNA levels were not changed in placentas of heterozygous or knockout DREAM-deficient mice compared to wild type littermates at ED13.5 as shown by quantitative real-time PCR (F). In addition, in the transgenic DREAM-over-expressing mouse strain JN1, no ectopic placental expression of DREAM could be detected as revealed by quantitative real-time PCR (G). Data are show as averaged values with standard deviations of a minimum of three litters per gestational day. No statistical differences were detected performing a 1-way ANOVA test followed by Bonferroni post-hoc test in GraphPad Prism 5.0. (TIF) [file pone.0051837.s003.tif]
