# Supplementary material for: DREAM Mediated Regulation of GCM1 in the Human Placental Trophoblast
Source: PLoS One. 2013 Jan 3;8(1):e51837. doi: 10.1371/journal.pone.0051837 (PMC3536794; doi:10.1371/journal.pone.0051837)
Supplement: File S1 — Supplementary Methods and Results . DREAM DNA methylation analysis indicates that this epigenetic mechanism is not responsible for de-repression of DREAM in sPE placentas. (DOC) [file pone.0051837.s004.doc]

Supplementary Methods

**DNA methylation analysis**

1 ug of genomic DNA was subjected to sodium bisulfite conversion and subsequently analyzed by Analytical Genetics Technology Centre in Toronto, Canada. The positive (completely methylated DNA) and negative (demethylated DNA) controls were used. The methylation status of the DREAM promoter region of preeclamptic patients (n=8) and aged-matched controls (n=8) was investigated in four sets of amplicons spanning almost 2000bp CpG island.

Supplementary Results

**DREAM DNA methylation pattern**

DREAM promoter contains a dense CG-rich CpG island, suggesting a potential role for methylation in regulation of its expression. Therefore we compared the methylation status of 92 CpG sites extending over 2000bp region upstream of the transcriptional start site of DREAM isoform in sPE and age-matched control placental samples. Each potential methylation sites in all of the analyzed samples exhibited low methylation ratesof 9% (40% range). No sites exhibited significant de-methylation (>10% difference) comparing sPE to normal tissues indicating that this epigenetic mechanism is not responsible for de-repression of DREAM in sPE placentas.
